# Microbial Diversity and Its Relationship to Physicochemical Characteristics of the Water in Two Extreme Acidic Pit Lakes from the Iberian Pyrite Belt (SW Spain)

**DOI:** 10.1371/journal.pone.0066746

**Published:** 2013-06-26

**Authors:** Esther Santofimia, Elena González-Toril, Enrique López-Pamo, María Gomariz, Ricardo Amils, Ángeles Aguilera

**Affiliations:** 1 Instituto Geológico y Minero de España, Madrid, Spain; 2 Centro de Astrobiología, Madrid, Spain; 3 Departamento de Fisiología, Genética y Microbiología, Universidad de Alicante, Alicante, Spain; 4 Centro de Biología Molecular Severo Ochoa, Universidad Autónoma, Madrid, Spain; Catalan Institute for Water Research (ICRA), Spain

## Abstract

The Iberian Pyrite Belt (IPB) hosts one of the world’s largest accumulations of acidic mine wastes and pit lakes. The mineralogical and textural characteristics of the IPB ores have favored the oxidation and dissolution of metallic sulfides, mainly pyrite, and the subsequent formation of acidic mining drainages. This work reports the physical properties, hydrogeochemical characteristics, and microbial diversity of two pit lakes located in the IPB. Both pit lakes are acidic and showed high concentrations of sulfate and dissolved metals. Concentrations of sulfate and heavy metals were higher in the Nuestra Señora del Carmen lake (NSC) by one order of magnitude than in the Concepción (CN) lake. The hydrochemical characteristics of NSC were typical of acid mine waters and can be compared with other acidic environments. When compared to other IPB acidic pit lakes, the superficial water of CN is more diluted than that of any of the others due, probably, to the strong influence of runoff water. Both pit lakes showed chemical and thermal stratification with well defined chemoclines. ***One particular characteristic*** of NSC is that it has developed a chemocline very close to the surface (2 m depth). Microbial community composition of the water column was analyzed by 16S and 18S rRNA gene cloning and sequencing. The microorganisms detected in NSC were characteristic of acid mine drainage (AMD), including iron oxidizing bacteria (*Leptospirillum, Acidithiobacillus ferrooxidans*) and facultative iron reducing bacteria and archaea (*Acidithiobacillus ferrooxidans, Acidiphilium, Actinobacteria, Acidimicrobiales, Ferroplasma*) detected in the bottom layer. Diversity in CN was higher than in NSC. Microorganisms known from AMD systems (*Acidiphilium*, *Acidobacteria* and *Ferrovum*) and microorganisms never reported from AMD systems were identified. Taking into consideration the hydrochemical characteristics of these pit lakes and the spatial distribution of the identified microorganisms, a model explaining their geomicrobiology is advanced.

## Introduction

The peculiar ecology and physiology of extremophiles and the environments in which they develop have interested microbiologists since their discovery. Their unusual properties make the exploration of their biotechnological potential promising [1], [2], [3], [4]. Environments below pH 3 are not abundant. They are mainly associated with two phenomena: volcanic and hydrothermal activities (e.g. hot sulfur springs, hot spots) or metal and coal mining activities [5]. This second case is especially interesting because, in general, the extremely low pH of the habitat is the consequence of microbial metabolism [6] and not a geophysical condition imposed by the system, as is the case in many other extreme environments (e.g. high temperature, ionic strength, radiation, or pressure).

In mining areas where sulfur-containing ores are brought to the surface, oxidation of sulfides by acidophilic chemolithotrophic bacteria often leads to the formation of highly acidic, metal-laden waters [7] known as Acid Mine Drainage (AMD). Conventional and molecular microbial ecology studies have shown that both sulfur- and iron-oxidizing bacteria, such as *Acidithiobacillus ferrooxidans* and *Leptospirillum ferrooxidans*, are present in the AMD ecosystems [6], [8], [9]. Members of these genera are frequently found in metal-rich acidic environments associated with metal sulfide leaching, i.e. hydrometallurgy operations or AMD generating systems [4], [10], [11].

The Iberian Pyrite Belt (IPB) hosts one of the world’s largest accumulations of mine wastes and AMDs [12], including Río Tinto, the only naturally occurring acidic river that has been described in scientific literature. IPB ores lack carbonates and are predominantly pyritic, fine-grained, and highly reactive [13], [14]. These characteristics favour the oxidation and dissolution of metallic sulfides, mainly pyrite, and the subsequent formation of AMDs.

Currently, there are more than 25 pit lakes between the provinces of Huelva and Seville (SW Spain) [15]. IPB pit lakes are normally acidic and present a wide range of sizes, ages, depths, and chemical characteristics. Most pose a serious environmental problem to the areas in which they are located. The low pH and high concentration of toxic heavy metals have limited the biological diversity of these lakes. Thus, the diversity of metazoan life is limited, and fish, amphibians, and mollusks are absent.

The geomicrobiology of the IPB is primarily known from studies of the Río Tinto [6], [10], [11], [16], [17], [18], [19], [20]. Our knowledge of microorganisms in the pit lakes in this area is far from complete. Most studies of the microbiology of the IBP have focused on the biological oxidation of iron and sulfur, whereas little is known of the biological reduction of these elements under the anaerobic conditions existing at the bottom of these lakes. To gather information about these biological processes, it is necessary to study efficient biotechnological processes, such as biomining or bioremediation. Recently, a biogeochemical study performed in the Cueva de la Mora pit lake in the IPB was published [21]. In this study metabolic activity was analyzed, but the study of microbial diversity was incomplete. However, each acid lake in the IPB presents unique characteristics. To our knowledge, this is the first complete report in which the physicochemical characteristics of the acid water in pit lakes from the IPB have been analyzed and correlated with their microbial diversity. Analysis of the prokaryotic and eukaryotic diversity of two different IPB pit lakes was carried out using multivariate statistical analysis to determine trends and infer possible interactions between microbial communities and physicochemical characteristics of the water.

## Materials and Methods

### Study Sites and Characteristics of the Pit Lakes

Two acidic pit lakes located on the IPB were analyzed, Lake Nuestra Señora del Carmen (NSC) and Lake Concepción (CN) (UTM coordinates NSC 110800; 4181909 and CN 176155; 4187355). Opencast exploitation of the NSC mine was abandoned in 1976 [22]. The pit was excavated at the bottom of a streambed near the Trimpancho stream confluence. A bypass channel was constructed in the mine to prevent pit flooding, but it is now filled with tailings. As a result, all freshwater runoff from the catchment area (270000 m^2^) flows into the pit. A containment wall regulates maximum lake levels to enable the transfer of water to the Trimpancho stream when lake levels rise. The pit lake dimensions are 110 m×80 m, with a depth of 34 m and a volume of ca. 79500 m^3^ [23].

The deposits of the CN mine were exploited by underground and opencast mining. The mine was abandoned in 1986 and is now completely flooded. The mining pit was excavated in a streambed and a bypass channel was constructed to prevent pit flooding. Now this channel is broken and freshwater runoff from a basin of 380000 m^2^ flows into the pit. The lake level is regulated by an exit through a mine adit, generating AMD, which flows into a small tributary of the Odiel river. The pit lake is also hydraulically linked to groundwater that circulates through underground mines. Three flooded shafts were explored in the mining area. The pit lake dimensions are 280 m×60 m, with a depth of 16 m and a volume of ca. 72500 m^3^ [24].

### Field Measurements and Sampling

Field measurements and water sampling were carried out in May 2009. No specific permits were required for the described field studies because the location is not privately-owned or protected in any way and field studies did not involve endangered or protected species. Depth measurements and vertical profiles of pH, redox potential (ORP), temperature (T), dissolved oxygen (DO), electric conductivity (EC), turbidity, photosynthetically active radiation (PAR) and chlorophyll-a (CHL-a) concentration were made with a Hydrolab® Datasonde S5 probe from the Hach Company.

The concentration of Fe(II) was measured by reflectance photometry with a Merck RQflex10 reflectometer and Reflectoquant® analytical strips. Nutrient concentration (N as NO_2_, N as NH_4_ and P as PO_4_) were measured with a Hanch-Lange DR 2800 Spectrophotometer.

Lake water samples were collected from different depths using an opaque, 2.2 L-capacity, BetaPlus® PVC bottle from Wildlife Supply Company®. All samples were filtered on site with 0.45 µm membrane filters from Millipore, stored in 125 mL polyethylene bottles, acidified with HNO_3_ (1 mL), and refrigerated at 4°C during transport. One liter samples for DNA extraction were collected and immediately filtered *in situ* through a Millex-GS Millipore filter (pore size, 0.22 µm; diameter, 50 mm), usually within 1 hr after the samples were collected. Filters were stored at −20°C until processing. Samples for microscopic observation were collected in sterile tubes, fixed with 2% of formaldehyde and kept at 4°C until further processing.

### Laboratory Water Analyses

Water samples were analyzed by Atomic Absorption Spectrometry (AAS, Varian SpectrAA 220 FS equipment) for Na, K, Mg, Ca, Fe, Cu, Mn, Zn and Al, by Inductively Coupled Plasma-Atomic Emission Spectrometry (ICP-AES, VarianVista MPX equipment) for Ni and S; and by Inductively Coupled Plasma-Mass Spectrometry (ICP-MS, Leco Renaissance) for V, Cr, Co, Cd, Sb, Ba, Tl, Th, and U. Sulfate was gravimetrically measured as BaSO_4_. The accuracy of the analytical methods was verified against certified water references (TM-27.3 and TMDA-51.3 from National Water Research Institute). Close agreement with the certified values was obtained for all metals. ^115^In was used as an internal standard for the calibration and measurements of the ICP-MS determinations. DOC was analyzed by a Shimadzu TOC-V CPH analyzer and PO_4_ by absorption UV-Vis spectrophotometry with an Alliance Integral Plus continuous flow autoanalyzer.

### Microscopy and Morphotype Identification

Identification of algae and heterotrophic protists was carried out down to the lowest possible taxonomic level by direct microscopic observation of different phenotypic features based on previous studies of the eukaryotic communities in acid environments [18], [19], [20]. A Zeiss Axioscope 2 microscope equipped with phase-contrast was used in this work.

### DNA Extraction, PCR Amplification and Sequencing

Fast DNA Spin kit for soil (Q-Bio Gene Inc., CA, USA) was used for DNA extraction according to the manufacturer’s instructions. Samples were washed five times with TE buffer (10 mM Tris HCl, 1 mM EDTA, pH 8) prior to DNA extraction. DNA was purified by passage through a GeneClean Turbo column (Q-Bio Gene Inc., CA, USA). The 16S and 18S rRNA genes were amplified according to previously described methodologies [6], [18] using the universal *Bacteria*-specific primers 27f and 1492r [25], [26], *Archaea*-specific primers 21f and 1492r [27], and *Eukarya*-specific 20f and 1800r primers [18]; PCR amplified genes were purified by GeneClean Turbo Column (Q-Bio Gene Inc., CA, USA) and cloned using the Topo TA Cloning Kit (Invitrogen, CA, USA). Primers used for sequencing were primers M13f and M13r recommended by TOPO TA kit. PCR products were directly sequenced using a Big-Dye sequencing kit (Applied Biosystem) according to manufacturer’s instructions.

### Phylogenetic Analysis

Sequences were analyzed using BLAST at the NCBI database (http://ncbi.nlim.nih.gov/BLAST) and added together with the most important BLAST hits to create a database of over 50000 homologous prokaryotic 16S rRNA primary sequences by using the ARB software package aligning tool [28]. Phylogenetic trees were generated using parsimony and neighbor-joining with a subset of 100 nearly full-length sequences (>1400 bp). Filters which excluded highly variable positions were used. Sequences obtained in this study have been deposited in the EMBL sequence database under accession numbers KC619546-KC619624.

An ARB-generated distance matrix was used as the input file to obtain distance-based operational taxonomic units (OTUs) and richness (DOTUR) [29], which assigns sequences to OTUs for every possible distance. Rarefaction analysis and the Chao1 non-parametric diversity estimator [30] were applied to the clone library in order to estimate how completely the library had been sampled and to extrapolate to total sequence diversity. Community structure was studied by estimating the similarity between communities based on membership and structure [29].

### Statistical Analysis: Multivariate Analysis

Data (dependent and independent factors) were analysed using a combination of constrained and unconstrained multivariate statistical methods in order to account for both total variation in the data and variation explainable by environmental data. Of the elements analyzed, we retained 20 environment parameters that had no missing values. We also used the pH, redox potential, electric conductivity, dissolved oxygen, PAR and temperature values measured in the field with probes. Canonical Correspondence Analysis (CCA) was used to compare the microbial community data with environmental data. It was conducted using OTUs of 16S rRNA genes acquired at different sampling depths. The significance of the first CCA axis and of all CCA axes combined, were tested using Monte Carlo permutation tests. CCA tests were performed by using the multivariate data analysis software CANOCO 4.5 (Microcomputer Power, Ithaca, NY, USA) [31]. The program CANODRAW 4.0 (in the Canoco package) was used for graphical presentation of ordination results.

## Results

### Pit Lakes Hydrochemistry

**Figure 1:**
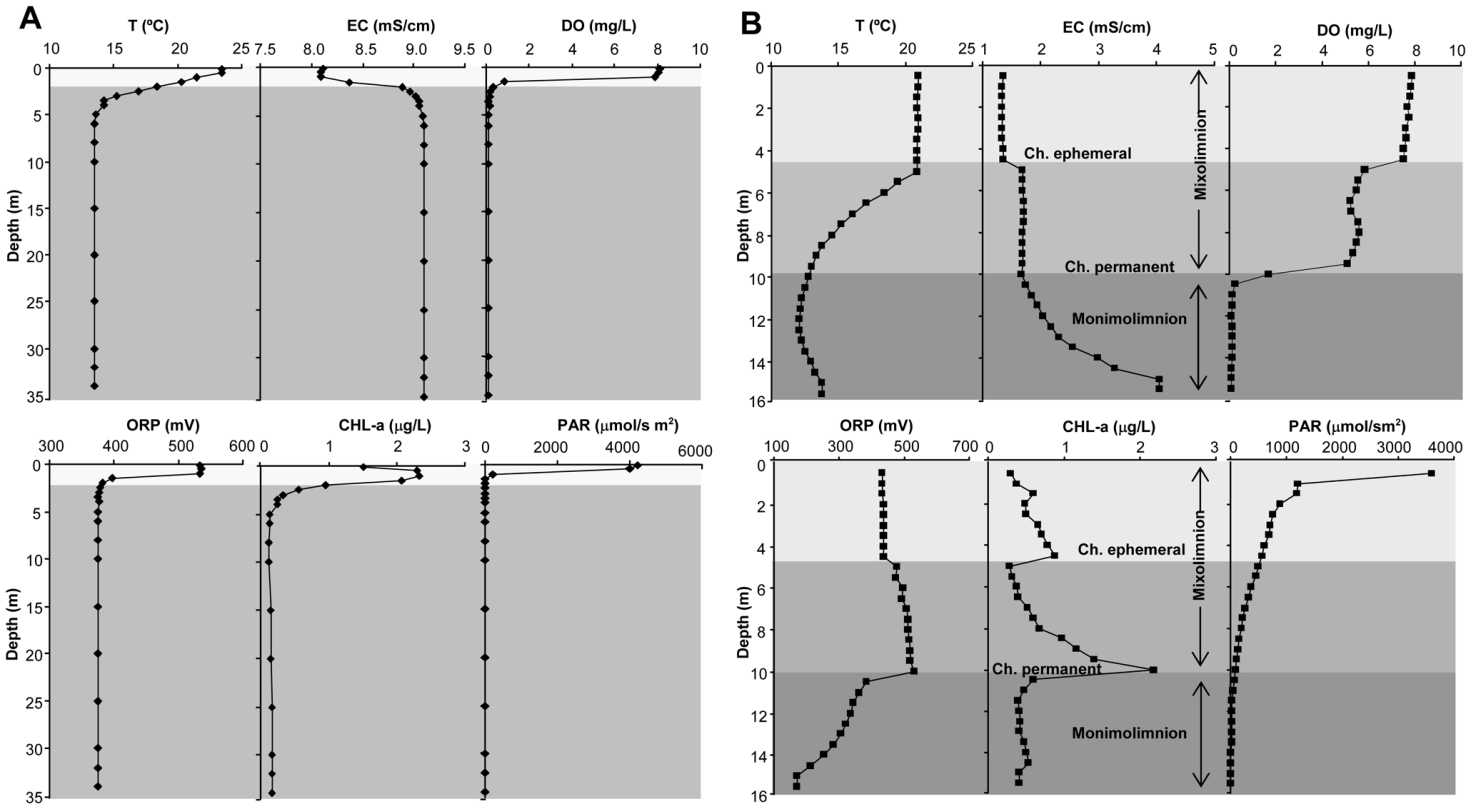
Water depth profiles. Temperature (T), electric conductivity (EC), dissolved oxygen (DO), redox potential (ORP), chlorophyll-a (CHL-a) and photosynthetically active radiation (PAR) in A) NSC pit lake and B) CN pit lake. Data obtained in May 2009. Ch. Chemocline.

The NSC pit lake water was acidic (pH ∼2.2) with high concentrations of sulfate (8.5 g/L), magnesium (1.0 g/L) and metals (Fe 760 mg/L, Al 190 mg/L, Mn 84 mg/L and Cu 29 mg/L; Table 1). The NSC pit lake normally shows chemical stratification; it is a meromictic lake. However, at the beginning of some dry winters, a period of mixing and total homogenization has been observed, temporarily transforming it into a holomictic lake. This process ended after intense episodes of rain, and the chemical stratification resumed as a result of the inflow of runoff water [23]. In May 2009, the pit lake showed a two-layer chemical and thermal stratification: a thin upper layer (2 m depth) and a denser, homogeneous bottom layer (from 3 to 35 m depth, Fig. 1A). High redox values (ORP ∼535 mV), corresponding to oxygen-saturated conditions, were measured in the surface layer, where Fe was predominantly oxidized (Table 1). In the chemocline PAR values were low, but sufficient to promote photosynthetic activity (Fig. 1A) in the first few meters of the water column despite intensely attenuated visible light (PAR) caused by the high concentration of dissolved solids [32], [33]. The chemocline appears to be the optimal place for phytoplankton growth since the highest measured nutrient (C, N, P) values were in the bottom layer (Table 1).

**Table 1:** Water chemical composition.

		Nuestra Señora del Carmen pit lake					Concepción pit lake						
Data	Unit	Upper layer	Bottom layer			Mixo-						Moni-	
		0 m	5 m	15 m	25 m	0 m		2 m	4 m	7 m	10 m	13.5 m	
**SO_4_^2-^**	g/L	7.6	8.1	8.5	8.5	0.393		0.465	0.481	0.732	0.797	1.87	
**Na**	mg/L	80.2	86.2	90	95	12.6		12.8	11.6	14.5	13.4	14.9	
**K**	mg/L	0.71	0.23	0.23	0.23	1.28		1.13	1.06	1.1	1.44	2.51	
**Mg**	mg/L	948	977	1024	985	44.6		44.9	45	65	69	112	
**Ca**	mg/L	420	449	441	440	37.6		38.3	38.7	51.5	52.5	72.6	
**Al**	mg/L	204	197	190	190	28		27.9	27.5	39.6	39.4	96.2	
**Cu**	mg/L	27.4	28.6	29.6	28.8	3.19		3.22	3.23	5.03	5.51	12.8	
**Fe(II)**	mg/L	0.9	338	338	353	3.1		2.6	0.7	0	3.7	495	
**Fe(III)**	mg/L	596	368	388	350	6.4		7.1	9.3	22.5	31.8	15	
**Mn**	mg/L	77.4	82.2	84.1	83.5	1.11		2.22	3.33	4.44	5.55	6.67	
**Zn**	mg/L	8.2	8.99	8.9	8.84	7.18		7.12	7.26	11.2	11.9	32.7	
**Ni**	µg/L	781	764	761	743	45		52	52	81	87	209	
**Co**	µg/L	645	672	666	672	248		235	241	358	373	592	
**Cr**	µg/L	67.5	67.7	67.8	67.8	<d.l.		<d.l.	<d.l.	<d.l.	<d.l.	<d.l.	
**Cd**	µg/L	12.5	13.0	13.2	13.1	19.9		18.8	18.7	30.8	35.7	73.6	
**Pb**	µg/L	6.2	13.3	13.3	12.6	36		36.3	34.5	43.9	53.9	122	
**DOC**	mg/L	1.90	2.79	2.52	2.41	<d.l.		<d.l.	0.66	0.63	0.89	0.88	
**PO_4_**	mg/L	0.22	0.31	0.39	0.39	<d.l.		<d.l.	<d.l.	<d.l.	<d.l.	0.11	
**NO_2_-N**	mg/L	<d.l.	<d.l.	<d.l.	<d.l.	<d.l.		<d.l.	<d.l.	<d.l.	<d.l.	<d.l.	
**NH_4_-N**	mg/L	0.024	0.119	0.114	0.075	0.023		n.a.	n.a.	0.027	0.092	0.336	
**PO_3_-P**	mg/L	0.137	0.161	0.150	0.149	<d.l.		n.a.	n.a.	<d.l.	<d.l.	0.059	

Major elements, selected trace element composition and nutrients of NSC and CN pit lakes. Data from May 2009. (n.a.) Not analyzed, (d.l.) Detection limit.

The CN pit lake was also acidic and showed relatively low concentrations of sulfate and metals (Fe, Al, Zn, Mn and Cu) (Table 1). The vertical profiles of physico-chemical parameters (Fig. 1B) and water chemistry obtained in May 2009 and in a recent study [24] showed a permanent chemical and seasonal thermal stratification (meromictic lake). The mixolimnion (upper layer) represented 90–95% of the total volume of the lake. This layer was oxygenated, less dense and ∼10 m deep, with pH between 2.5–2.7 and EC between 1.3–1.7 mS/cm. The monimolimnion (lower layer) was anoxic, denser, and extends from 10 m to 16 m; the pH was between 2.5–3.5, the EC was from 1.7–4.5 mS/cm, and the Fe concentration was up to 500 mg/L (mainly Fe(II)). A permanent chemocline that changes its position (in depth) according to an annual cycle was located between the layers. A shallow chemocline was also observed during episodes of heavy rain due to the dilution of the upper layer [24]. Thermal and chemical stratification can explain the subsaturation levels in the intermediate layer (between 2–6 mg/L), which is shown in the DO data.

### Microbial Diversity in NSC Pit Lake

**Figure 2:**
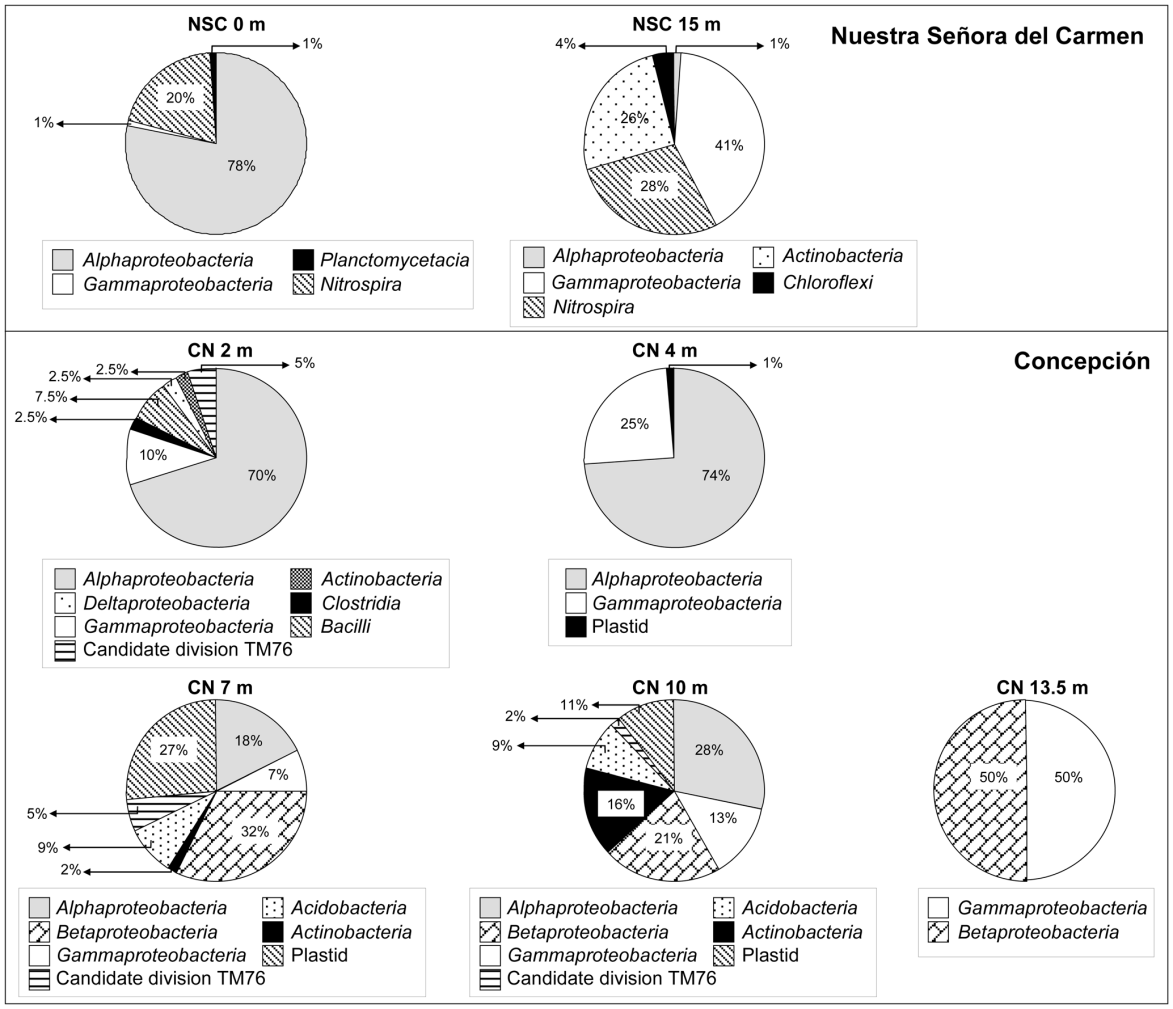
Abundance of bacteria from NSC and CN pit lakes. Relative abundance (in percent) of OUTs in bacterial groups is shown for each depth.

Bacterial 16S rRNA gene clone libraries: To correlate microbial diversity with observed hydrochemical conditions, water was sampled at 0 m depth in the upper layer and at 15 m depth in the bottom layer. After removing chimerical assemblies, a total of 160 bacterial sequences were selected for phylogenetic analysis. Rarefaction analysis and the Chao 1 estimator indicated that the diversity in the library was low enough to ensure sufficient coverage in the screening (Fig. S1A and Table S1in File S1). Members of the *Alphaproteobacteria*, *Gammaproteobacteria* and *Nitrospira* phyla were present at both depths. Additionally, *Planctomycetes* were detected in the upper layer and *Actinobacteria* and *Chloroflexi* were present at 15 m. The phylogenetic groups detected at different depths are shown in Fig. 2 and Table S2 in File S1. In the upper layer, almost 80% of the identified sequences belonged to the *Alphaproteobacteria* class and the most abundant phylotypes were closely related to *Acidiphilium*. Only 20% of the sequences detected at the surface were related to the *Nitrospira* class, all clustering within the genus *Leptospirillum*. In contrast, at 15 m depth, sequences related to *Gammaproteobacteria* (41%), clustered within the species *At. ferrooxidans*. 28% of the sequences found at this depth were related to *Nitrospira* class, with *Leptospirillum* being the most abundant genus, and 26% of the sequences clustered within the *Actinobacteria* class. At 15 m sequences related to the *Chloroflexi* and *Acidisphaera* genus (ca. 4% and 1% respectively) were also detected.

Archaeal 16S rRNA gene clone libraries: Sequences belonging to the *Archaea* domain were found only at 15 m depth (Table S2 in File S1), and all of them were related to the *Euryarchaeota* phylum, specifically to the *Thermoplasmata* class, which is closely related to uncultured archaea detected in extreme acidic environments (AY789596, EF600910).

Eukaryotic diversity: Eukaryotic 18S rRNA genes could only be amplified from the surface layer sample and results of the clone library are presented in Table S2 (in File S1). All identified sequences were related to the genus *Chlamydomonas.* Microscopic observations also showed the presence of filamentous species related to the genera *Zygnemopsis* and *Klebsormidium* as well as diatoms belonging to the genus *Pinnularia* in the surface sample, which were not detected by molecular techniques.

### Microbial Diversity in CN Pit Lake

Bacterial 16S rRNA gene clone libraries: CN pit lake samples were obtained from 2, 4, 7, 10, and 13.5 m depth. After removing chimerical assemblies, a total of 350 bacterial sequences were selected for phylogenetic analysis. Rarefaction analysis reached an asymptote for every sampling depth with the exception of 13.5 m (Fig. S1B in File S1). This was confirmed by the Chao1 estimator (Table S1 in File S1). The phylogenetic groups detected at the different stations are reported in Fig. 2 and in Table S3 (in File S1). At 2 m depth, 70% of the sequences were related to the *Alphaproteobacteria* class, with the most abundant phylotypes being closely related to *Acidiphilium* and *Acidisphaera* genera. Species belonging to *Gammaproteobacteria* and *Bacilli* classes represented ca. 20% of the remaining sequences found at this depth, with *Legionella* and *Bacillus* being the most abundant genera detected.

Biodiversity was greatly reduced at 4 m. Most of the sequences (74%) were related to *Alphaproteobacteria* class, with *Acidiphilium* being the most abundant genus. *Gammaproteobacteria* of the genus *Erwinia* represented the remaining sequences detected. At 7 m depth the number of species found increased again, with the most abundant being related to an uncultured betaproteobacterium (accession number DQ480476) (32%) related to “*Ferrovum myxofaciens”* [34] and an uncultured eukaryotic alga (27%) represented by its chloroplast. Almost 20% of the sequences were related to *Alphaproteobacteria* phylotypes from the genera *Acidocella* and *Acidisphaera*. Species of the genera *Acidobacterium* (9%) and *Legionella* (7%) were also found.

At 10 m depth biodiversity was also high and similar to the 7 m sample in terms of identified phylotypes, although the percentages were different. The main changes were related to the increase in members of the *Actinobacteria* class, from 2% at 7 m to 16% at 10 m depth. Sequences clustering with *Halomonas (Gammaproteobacteria)* were also identified for the first time in this type of acidic environment. Finally, the sample from 13.5 m depth showed the lowest diversity. Half of the detected sequences were related to *Thiobacillus* genus and an uncultured bacterium TRA3-20, and the remaining 50% of the clones clustered with the *Acidimicrobiaceae* family.

Eukaryotic diversity: Two chloroplast sequences were detected, both related to uncultured microorganisms. The first, found at 4 m, 7 m and 10 m depth, was related to the sequence HQ420121 (99% similarity), previously detected in an acid mine drainage site [35]. The second was related to an uncultured rhodophyte (89–90% similarity) and was detected at 7 m and 10 m depth (Table S3 in File S1). In addition, a eukaryotic18S rRNA gene clone library from each sampling site was constructed. Positive results were found at all depths with the exception of 13.5 m. Sequences related to the genus *Chlamydomonas* were detected only at 2 m. The remaining 18S rRNA gene sequences were related to *Ochromonas* (*Chrysophyceae,* 99% similarity) and were detected at 2 m, 4 m, 7 m and 10 m depth.

### Multivariate Analyses

**Figure 3:**
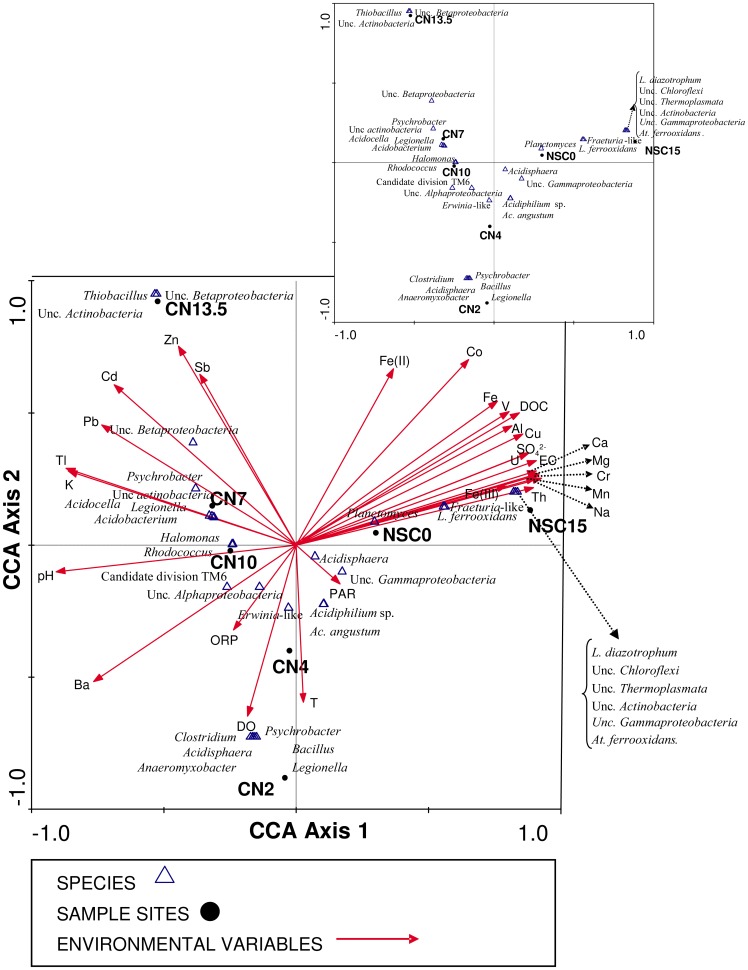
CCA triplot correspondence analysis based on variance of species (OTUs) with respect to environmental data. The eigenvalues for each axis generated by CCA indicate how much of the variation seen in species data can be explained by that canonical axis. 50% of the correlation between OTUs, samples sites and environmental data was explained by two axes. The presence or absence of data were used for bacterial, archaeal and eukaryotic OTUs. Different OTUs are represented by species names and triangles. Environmental variables used in the analysis are shown by arrows. Sampling sites are indicated by dots and the station name (CN2, CN4, CN7, CN10, CN13.5, NSC0 and NSC15).

Environmental data were transformed using ln(x+0.1) and normalized to zero mean and unit variance. Canonical Correspondence Analysis (CCA) was used to correlate microbial community data with environmental data. It was conducted using OTUs of 16S rRNA genes acquired at different sampling depths. The CCA triplot in Figure 3 summarizes the results and shows the correlation between environmental variables, samples, and OTUs. Samples were plotted in different areas of the diagram depending on their environmental characteristics. The CCA technique generates an ordination diagram in which axes are created by a combination of environmental variables [36]. The eigenvalues for each axis generated by CCA indicate how much of the variation seen in species data can be explained by that canonical axis. In this case, 50% of the correlation between OTUs, sampling sites and environmental data was explained by two axes.

The first axis shows the highest positive correlation among a wide number of variables (major ions SO_4_^2–^, Mg, Ca, Na, heavy metals: Fe, Al, Cu, Mn and trace elements: Cr). In contrast, pH showed the highest negative correlation with this axis. Samples from the different pit lakes were also discriminated by this axis. Thus, NSC samples are located near the positive area of the axis, while samples from CN fall in the negative area of the same axis, meaning that NSC pit lake is more acidic and contains more dissolved solids than CN pit lake.

The right-hand portion of the first axis was predominantly occupied by bacterial and archaeal OTUs detected in samples from NSC pit lake (Fig. 3), such as *Leptospirillum* spp., *At. ferrooxidans*, *Acidisphaera*, uncultured *Actinobacteria* and uncultured *Thermoplasmata*. *L. ferrooxidans* was frequently found at both depths (0 and 15 m), while other species such as *At. ferrooxidans* or uncultured *Chloroflexi* were found only in the deep sample (NSC 15 m) (Fig. 3).

Zn and Fe(II) showed the highest positive correlation with the second axis, while dissolved oxygen and temperature were the parameters with the highest negative correlations. This second axis was able to discriminate among the different sampling depths in the CN pit lake water column. Both superficial samples (CN 2 m and CN 4 m) are near the negative area of the axis, associated with high levels of dissolved oxygen levels and high temperatures. In this area, OTUs correlating with *Erwinia*, *Acidisphaera* or *Acidiphilium* spp. were also found (Fig. 3). On the other hand, the deepest sample (CN 13.5 m), located in the positive area of the axis, is anoxic and also showed the lowest temperature and the highest concentrations of Zn and Fe(II). This sampling site showed a peculiar microbiology that was different from the other CN pit lake samples. OTUs such as *Thiobacillus* or the iron oxidizer “*Ferrovum myxofaciens*” [34] were found. The highest biodiversity was found at intermediate depths (CN 7 m and CN 10 m) in the water column. This area of the plot is predominantly occupied by bacterial OTUs related to *Psychrobacter, Legionella, Acidobacterium* and *Acidocella* (Fig. 3).

## Discussion

The two main objectives of this work are to investigate the microbial diversity in two hydrochemically different extreme acidic pit lakes and to determine the possible impact of physico-chemical parameters on the microbial community assemblage. Although other studies have been performed on acidic IPB environments, such as Río Tinto, to our knowledge this is the first report in which the physico-chemical parameters of acidic pit lake water are analysed with regard to their associated microbial communities. This study demonstrates that these lakes, associated with mining of sulfidic ore, are inhabited by a diverse microbial community that differs from the communities usually reported in the IPB. Prokaryotic sequences related to the genus *Erwinia*, *Legionella* or *Halomonas*, never described before in this area, were detected.

**Figure 4:**
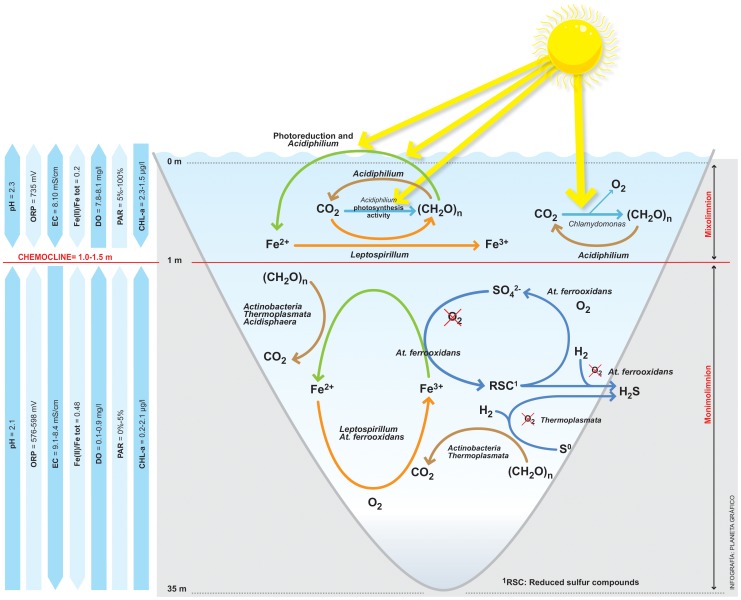
Geomicrobiological model of the NSC pit lake. The model shows the roles of the different microorganisms identified in the ecosystem. Microorganisms are shown associated with their roles in the iron and carbon cycles.

Pit lakes NSC and CN were chosen because of their different physicochemical characteristics and their structural differences. In May 2009, NSC pit lake was clearly chemically and thermally stratified, showing two different layers. The chemocline of this lake was closer to the surface (between 1–3 m depth), when compared to other meromictic lakes along the IPB [15]. Usually the bottom layer of meromictic lakes is governed by anoxic and low redox conditions coupled with bacterial reduction of Fe(III), which causes dissolved Fe to be normally found as Fe(II) [37], [38], [39], [40]. In contrast, during this study the bottom layer of the NSC pit lake exhibited a slightly reduced environment (ORP ∼440 mV) and similar concentrations of Fe(II) and Fe(III) (ca. 300 mg/L, Table 1). This atypical distribution of the iron redox pair was previously noted in other pit lakes [39], [41]. Earlier studies [23], [42] revealed changes in the Fe(II)/Fe(III) ratio (decreases or increases) of the NSC bottom layer, which were the result of lake dynamics. During mixing, dissolved oxygen (DO) descends from the surface and promotes an increase in biological iron oxidation processes, which in turn leads to a decrease in the Fe(II)/Fe(III) ratio. When stratification in the pit lake lasted for several months, an increase of Fe(II)/Fe(III) ratio values resulting from bacterial reduction processes was measured [42]. The lake dynamics in NSC seem to control the distribution of iron redox species through several winter mixing processes [23]. As a result, the transferred water mass provided dissolved oxygen to the bottom of the lake. This oxygen, together with the presence of iron oxidizing bacteria (mainly *L. ferrooxidans*) was responsible for the presence of Fe(III) (Fig. 4). The upper layer was less diverse bacteriologically than the lower one because most of the sequences detected were related to *Leptospirillum* spp. and *Acidiphilium* spp. This association is characteristic of AMD environments. *Leptospirillum* is an iron oxidizing bacterium, and *Acidiphilium* is responsible for removing organic compounds, which are toxic for *Leptospirillum* [9]. On the other hand, *Acidiphilium* reduces iron even in the presence of oxygen (up to 60% of DO [9]).

The bottom layer of the NSC pit lake showed the highest diversity level. This could be explained by the existence of diverse potential energy sources (Fe(II), reduced sulfur compounds, organic matter) available at lower depths (Table 1). In this case, the detected *Acidithiobacillus* spp. and members of the *Acidimicrobiacea* family, both facultative iron reducing bacteria under anoxic conditions [43] (Fig. 4), could be responsible for the increase in the Fe(II)/Fe(III) ratio in this layer when the pit lake is chemically stratified (normally ten to eleven months per year), while *Leptospirillum*, *Acidithiobacillus* and some *Acidimicrobiaceae* could oxidize iron in the presence of oxygen [9] during times when stratification breaks down and the water column overturns (Fig. 4).

A sulfur cycle must also be operating at NSC. Sulfur could be oxidized by *Acidithiobacillus* spp. when oxygen is present [9]. On other hand, sulfur is the energy source used by *At. ferrooxidans* during anaerobic respiration [9]. In addition, *At*. *ferrooxidans* (demonstrated in one strain only) could grow anaerobically using hydrogen as electron donor and sulfate as an electron acceptor [44]. *Thermoplasmata* could grow heterotrophically using hydrogen as electron donor and elemental sulphur as electron acceptor [9]. Sulfate-reducing bacteria were not detected in this system, probably due to organic acid toxicity at low pH or the inhibition caused by hydrogen sulfide [9].

Sequences closely related to *Chloroflexi* have been detected in acid mine drainage environments [45], [46], [47]. Several novel *Chloroflexi* have been reported to occur at high frequency and diversity ([48], but mostly unpublished). To our knowledge, *Chloroflexi* is a class that does not contain acidophilic or Fe(II) oxidizing members, and their potential role in the NSC system is still unclear. However, these bacteria could play an active biogeochemical role in these environments, especially in anaerobic sites.

In contrast, Fe(II) concentrations in the upper layer of the NSC pit lake were low (0.9 mg/L), compared to Fe(III) concentrations (596 mg/L). At the surface, *Acidiphilium*, together with photoreduction of Fe(III), could regenerate Fe(II), although it would be rapidly oxidized by *Leptospirillum* [41], [49], [50], [51] (Fig. 4).

In the NSC pit lake the DOC values were always low when compared with neutral natural lakes; for this reason lake NSC can be classified as oligotrophic, although these values are similar to those that other authors list for lakes consisting of mine water [52], [53]. DOC concentrations were higher in the bottom layer (2.65–5.75 mg/l) than in the upper one (0.79–3.28 mg/l; data not shown).

In the chemocline the detected high chlorophyll values (Fig. 1A) can be correlated with the *Chlamydomonas*, which we identified. In this layer, measured PAR values were low (between 14–235 µmol/s·m^2^), but sufficient to activate photosynthesis in acidophilic species [54]. Inorganic carbon (data not measured), nitrogen (as NH_4_ 0.024–0.027 mg/L, data not shown; the N concentrations as NO_2_ were below the detection limit), and phosphorus (values as PO_4_ 0.061–0.137 mg/L data not shown) limit phytoplanktonic productivity in mine pit lakes. The phosphorus measurements in the superficial layer were below the detection limit, probably due to its adsorption by schwertmannite [55], which has been identified in this layer of the pit lake [23].

**Figure 5:**
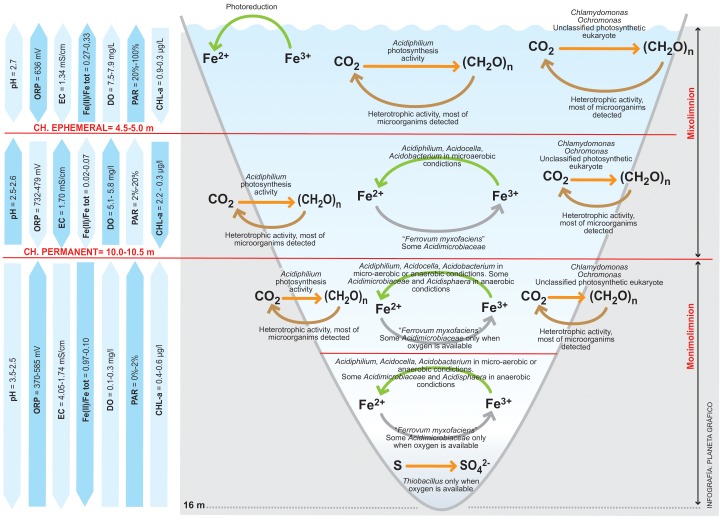
Geomicrobiological model of the CN pit lake. The model shows the roles of the different microorganisms identified in the ecosystem. Microorganisms are shown associated with their roles in the iron, sulfur and carbon cycles.

In contrast, microbial communities in the CN pit lake were diverse (Fig. 5). Although we identified microorganisms associated with AMD, we could not detect iron oxidizing bacteria characteristic of AMD systems, such as *Leptospirillum* spp. or *At. ferrooxidans*. In this pit lake, low dissolved iron concentrations seem to be responsible for the absence of these microorganisms. Only where dissolved iron concentrations increased with depth, were iron oxidizing *Betaproteobacteria* related to “*Ferrovum myxofaciens*” [34] mostly found. The AMD-associated microorganisms that we detected were mostly related to heterotrophic bacteria, mostly facultative iron-reducing bacteria (i.e. *Acidiphilium*, *Acidobacteria* or members of the *Acidimicrobiaceae* family) (Fig. 5). This particular microbial assemblage could be the result of the low concentrations of sulfate and heavy metals found in this water, as compared to other acidic pit lakes or AMD waters. Microorganisms rarely detected in AMD were also found in this pit lake, such as *Legionella*, *Erwinia* and *Halomonas*. The development of a mixolimnion in the CN pit lake seems to be the result of extensive inflow of freshwater (mainly runoff [24]). This factor could explain the presence of these microorganisms. Although it is difficult to find a specific role for these bacteria, most of them have been previously detected in mining associated environments, acidic soils and heavy-metals rich ecosystems. For example, a moderately halophilic arsenite-oxidizing *Halomonas* was isolated from acidic soil from a gold-mine [56] and was identified by molecular methods in a copper mine wasteland [57]. *Legionella* has been detected in acid mine drainages and an acidic biofilm community in Yellowstone National Park [58], [59]. Its detection has been explained by the presence of eukaryotes, which often host *Legionella* as endosymbionts [58], [60]. In the case of CN pit lake, *Legionella* could be hosted by *Chlamydomonas* and *Ochromonas*. Likewise, *Rhodococcus* species have also been detected in mining areas associated with organic sulfur bioleaching processes [61], [62].

Mixolimnion water of the CN pit lake is one of the most diluted (Table 1) of acidic lakes of the IPB [51], [15]. Due to low dissolved element concentrations (e.g. Fe, Al, Zn, Mn, Cu), PAR values were measured at depths down to 12 m (Fig. 1), promoting the development of eukaryotic population (up to 2 µg/L of CHL-a at 10 m depth). The highest chlorophyll-a values were observed in the permanent chemocline, since its growth depends on parameters such as light and temperature, but also on nutrients, which showed higher values in the monimolimnion (N and P values; Table 1). In the shallower layer, a total Fe concentration of 9.5 mg/L was measured, of which 3.5 mg/L were Fe(II), suggesting photoreducing activity of dissolved Fe(III), which has been previously measured in this type of acidic pit lake [33], [41], [49], [50], [51]. On the other hand, hydrochemical characteristics of the monimolimnion were similar to the mine groundwater that floods underground mine sites.

The particularly high microbial diversity found in the surface layer of the CN pit lake could also be explained by the inflow of seasonal freshwater. On other hand, the highest diversity was detected at 10 m depth, which coincides with the permanent chemocline and is considered as a transition zone characterized by higher variation in nutrients and chemical elements. Finally, the deepest part of the lake (the monimolimnion) showed a special microbiological composition and hydrochemical conditions, caused by groundwater inflow from shafts and flooded galleries through the bottom of the lake, which is very similar to water from AMDs.

CCA analysis confirmed that both pit lakes are different, although in both cases the correlation between hydrochemistry and diversity is clear. Thus, NSC microbial diversity is more related to heavy metals and sulfate concentration. On other hand, elements such as Zn, Cd or Pb, as well as physicochemical parameters such as pH or ORP, are more relevant with respect to microbial diversity in the CN pit lake. NSC microorganisms correlated mainly with iron, specifically Fe(III). In general, the species found in the NSC pit lake were iron oxidizing or facultative iron reducing bacteria [43]. The main cycle operating in this pit lake is the iron cycle [6], [9], [10]. It is also clear that there are two different environments, corresponding to the upper and the lower layers of the NSC pit lake (Fig. 4). Both are characterized by the presence of heavy metals, but at the surface iron oxidizing bacteria such as *L. ferrooxidans* were predominant, while at 15 m depth facultative anaerobic and iron reducing bacteria such as *At. ferrooxidans* were present. CCA analysis supports that, in the upper layer, the dominant metabolisms are based on iron oxidation while in the bottom layer iron reduction in combination with iron oxidation (an active iron cycle) are the dominant metabolisms.

In the case of CN, the correlation between microbial diversity and hydrochemical parameters was less evident, since the iron or sulfur cycles are not as dominant in this pit lake. Thus, the microorganisms detected are not clearly involved in a particular cycle (Fig. 5). Correlation between OTUs and heavy metals is not as strong as in NSC. Additionally, the lower heavy metal concentration makes this lake a less extreme ecosystem. Some OTUs detected are commonly found in AMD waters, but always associated with less extreme environmental conditions. Thus, *Acidocella* or *Acidobacterium* were found in this lake when the pH was slightly less acidic.

Interestingly, the *Acidiphilium* genus was found in both pit lakes. OTUs corresponding to *Acidiphilium* species were highly positively correlated with PAR in both pit lakes (Fig. 3). This correlation could be explained in terms of *Acidiphilium* species that have been described as aerobic zinc-bacteriochlorophyll-containing bacteria capable of anoxygenic photosynthesis [63]. Although the ecological role of this metabolic capability under aerobic conditions is not known, ^14^CO_2_ uptake by *Acidiphilium* species is clearly enhanced by light [64]. *Acidiphilum* was the most frequently represented genus at the surface of NSC, but was nearly absent at a depth of 15 m. In the CN pit lake, *Acidiphilium* was also highly represented at 2 and 4 m, where PAR was higher. Although most *Acidiphilium* species are obligate heterotrophs [9], their natural habitats seem to be oligotrophic environments with low concentrations of organic matter. The ability of *Acidiphilium* to perform photosynthesis with Zn-BChl *a* may be an advantage for developing in such oligotrophic acidic environments [63].

Furthermore, CCA supported the peculiar characteristic of samples from the deepest part of CN (13.5 m). This depth is within the anoxic monimolimnion, in which the detected OTUs corresponded to uncultured iron reducing bacteria previously associated with AMD [9]. The distribution of these bacteria has a highly positive correlation with Zn, indicating that this metal is relevant in their metabolism.

### Conclusions

The characterized NSC and CN pit lakes from the IPB represent two different environments. Based on a correlation between physicochemical and environmental parameters, we are able to propose a geomicrobiological model for the NSC pit lake. The bacterial assemblages found in this ecosystem seem to be dominated by species mainly involved in the iron cycle. OTUs related to iron-oxidizing bacteria, such as *L. ferrooxidans*, were detected at the surface of the lake. Other microorganisms, such as *Acidiphilium* were also numerous at the surface. Iron photoreduction was also high at the surface of the lake, and was a potential contributor to Fe(II) regeneration. In the deepest layers of the lake, microorganisms such as *At. ferrooxidans*, *Thermoplasmata*, species related to acidophilic actinobacteria able to oxidize or reduce iron (depending on the presence or absence of oxygen) were identified. The presence of these organisms suggests an increase in the level of physiologically versatile organisms capable of obtaining energy under variable redox conditions in this part of the water column. The identification of iron-oxidizing species of *Leptospirillum* suggests that iron oxidation is also possible. The predominance of iron oxidizing or reducing reaction depends on the availability of oxygen, which is introduced into the lake during the winter mixing. As a result of this variation in oxygen availability in the water column, the average [Fe(II)]/[Fe(III)] ratio that we detected was ca. 1. At 15 m depth, PAR values were non-existent and photosynthetic eukaryotes were absent.

In contrast, we found the CN pit lake to be a heterotrophic system. Facultative iron reducing bacteria and some iron oxidizing bacteria (predominantly *Betaproteobacteria*) were present, indicating the importance of the iron cycle, especially in the deepest part of the pit lake. At a depth of 13.5 m we detected a different system in which we found microorganism capable of promoting an iron cycle and sulfur oxidation. Since oxygen was not detected at this depth, these sulfur and iron oxidizing bacteria must be inactive except during times of intermittent oxygenation.

## Supporting Information

File S1 (DOC)
