# Supplementary material for: Microbial Diversity and Its Relationship to Physicochemical Characteristics of the Water in Two Extreme Acidic Pit Lakes from the Iberian Pyrite Belt (SW Spain)
Source: PLoS One. 2013 Jun 26;8(6):e66746. doi: 10.1371/journal.pone.0066746 (PMC3694112; doi:10.1371/journal.pone.0066746)
Supplement: File S1 — (DOC) [file pone.0066746.s001.doc]

**Figure S1.** Rarefaction analysis for 16S rRNA genes clones obtained from A) NSC pit lake and B) CN pit lake. Sequences were grouped into OTUs based on 97% sequence similarity and curves were calculated using DOTUR. Error bars represented the 95% CI.

**B**

**A**

**Table S1.** Number of OTUs obtained from NSC and CN pit lakes and Chao richness estimated.

| **NSC** | | | |
| --- | --- | --- | --- |
| Sampling depth | Observed number of OTUs | Chao1 | Shannon index |
| 0 m | 6 | 9 | 1.149 |
| 15 m | 14 | 19 | 1.924 |
| **CN** | | | |
| Sampling depth | Observed number of OTUs | Chao1 | Shannon index |
| 2 m | 13 | 18 | 2.059 |
| 4 m | 4 | 4 | 1.045 |
| 7 m | 11 | 14 | 1.839 |
| 10 m | 20 | 20 | 2.381 |
| 13.5 m | 4 | 5 | 1.455 |

**Table S2**. Phylogenetic affiliation and number of sequences obtained from clones from NSC pit lake. Closest relatives and their accession number are given.

| Affiliation (phylum/class/family/genus) and metabolic relationship with the iron and sulfur cycles | | | | | | | | | | | N^1^ (%) | Closest relative | |
| --- | --- | --- | --- | --- | --- | --- | --- | --- | --- | --- | --- | --- | --- |
|  |  |  |  |  |  |  |  |  |  |  |  | GeneBank Acc. | ID^2^ (%) |
| Nuestra señora Del Carmen 0 m | | | | | | | | | | | | | |
| *Proteobacteria* | | | | | | | | | | |  |  |  |
|  | | *Alphaproteobacteria* | | |  | | | | | | 64(78) |  |  |
|  | | | *Acetobacteraceae* |  | | *Acidiphilium* IRB^3^ | | |  | | 55 | DQ303265 | 98 |
|  | | |  |  | | *Acidiphilium* IRB | | |  | | 8 | EF556242 | 99 |
|  | | |  |  | | *Acidisphaera* IRB | | |  | | 1 | EF446227 | 99 |
|  | | *Gammaproteobacteria* | | | | |  | | | | 1(1) |  |  |
|  | | | Unclass. *Gammaproteobacteria* | | | Uncultured bacterium | | | |  | 1 | EU370277 | 99 |
| *Nitrospirae* | | | | | | | | | | |  |  |  |
|  | | *Nitrospira* | | | | |  | | | | 16(20) |  |  |
|  | | | *Nitrospiraceae* | | | *Leptospirillum* IOB^4^ | |  | | | 16 | EU372652 | 99 |
| *Planctomycetes* | | | | | | | | | | |  |  |  |
|  | | *Planctomycetia* | | | | | | | | | 1(1) |  |  |
|  | | | *Planctomycetaceae* | | | Uncultured bacterium | | | | | 1 | DQ906078 | 98 |
| *Chlorophyta* | | | | | | | | | | |  |  |  |
|  | | *Chlorophyceae* | | | | | | | | | 21(100) |  |  |
|  | | | *Chlamydomonadaceae* | | | *Chlamydomonas* | | | | | 21 | AY082979 | 99 |
| Nuestra señora Del Carmen 15 m | | | | | | | | | | | | | |
| *Proteobacteria* | | | | | | | | | | |  |  |  |
|  | | *Alphaproteobacteria* | | | | | | | | | 1(1) |  |  |
|  | | | *Acetobacteraceae* | | | *Acidisphaera* IRB | | | | | 1 | HM124396 | 97 |
|  | | *Gammaproteobacteria* | | | | | | | | | 32(41) |  |  |
|  | | | *Acidithiobacillaceae* | | | *Acidithiobacillus* IRB, IOB, SOB^5^, SRP^6^ | | | | | 28 | EF446219 | 99 |
|  | | | Unclass. *Gammaproteobacteria* | | | Uncultured bacterium | | | | | 1 | DQ480485 | 97 |
|  | | | Unclass. *Gammaproteobacteria* | | | Uncultured bacterium | | | | | 2 | FJ228294 | 96 |
|  | | | *Xanthomonadaceae* | | | WJ2 cluster IRB (Related to *Frateuria*) | | | | | 3 | EU370273 | 98 |
| *Nitrospirae* | | | | | | | | | | |  |  |  |
|  | *Nitrospira* | | | | |  | | | | | 22(28) |  |  |
|  | | | *Nitrospiraceae* | | | *Leptospirillum* IOB^4^ | | | | | 7 | DQ328620 | 98 |
|  | | |  | | | *Leptospirillum* IOB | | | | | 15 | EU372638 | 99 |
| *Actinobacteria* | | | | | | | | | | |  |  |  |
|  | *Actinobacteria* | | | | |  | | | | | 20(26) |  |  |
|  | | | *Acidimicrobiaceae* | | | Uncultured bacterium | | | | | 7 | AB254795 | 99 |
|  |  | | | | | Uncultured bacterium | | | | | 1 | AB254787 | 99 |
|  |  | | | | | Uncultured bacterium | | | | | 1 | DQ450881 | 99 |
|  |  | | | | | *Ferrimicrobium* IRB, IOB | | | | | 1 | AB254793 | 99 |
|  |  | | | | | Uncultured bacterium | | | | | 10 | AF523914 | 99 |
| *Chloroflexi* | | | | | | | | | | | 3(4) |  |  |
|  |  | | | | | Unclassified *Chloroflexi* | | | | | 3 | Fj228239 | 99 |
| *Euryarchaeota* | | | | | | | | | | |  |  |  |
|  | *Thermoplasmata* | | | | |  | | | | | 3(100) |  |  |
|  | | | *Thermoplasmataceae* | | | Uncultured archaeon. Could be IOA^7^, SRP | | | | | 1 | AY789586 | 94 |
|  |  | | | | | Uncultured archaeon. Could be IOA, SRP | | | | | 2 | EF600910 | 99 |

^1^ Relative abundance of clones and percentage, ^2^ Percentage of similarity, ^3^ IRB: Iron Reducing Bacteria; ^4^ IOB: Iron Oxidizing Bacteria; ^5^SOB: Sulfur Oxidizing Bacteria; SRP: Sulfur reducing prokaryotes; ^7^ IOA: Iron Oxidizing Archaea

**Table S3**. Phylogenetic affiliation and number of sequences obtained from clones from CN pit lake. Closest relatives and their accession number are given.

| Affiliation (phylum/class/family/genus) and metabolic relationship with the iron and sulfur cycles | | | | | | | | | | | | | | | N^1^ (%) | Closest relative | |
| --- | --- | --- | --- | --- | --- | --- | --- | --- | --- | --- | --- | --- | --- | --- | --- | --- | --- |
|  |  |  |  |  |  |  |  |  |  |  |  |  |  |  |  | GeneBank Acc. | ID^2^ (%) |
| Concepción 2 m | | | | | | | | | | | | | | | | | |
| *Proteobacteria* | | | | | | | | | | | | | | |  |  |  |
|  | *Alphaproteobacteria* | | | | | | | |  | | | | | | 28(70) |  |  |
|  | | *Acetobacteraceae* | | | | | |  | | *Acidiphilium* IRB^3^ | | |  | | 1 | JF737921 | 99 |
|  | |  | | | | | |  | | *Acidiphilium* IRB | | |  | | 12 | HM745451 | 99 |
|  | |  | | | | | |  | | *Acidisphaera* IRB | | |  | | 12 | HQ674805 | 98 |
|  | |  | | | | | |  | | *Acidisphaera* IRB | | |  | | 1 | JF737897 | 95 |
|  | | Unclass. *Alphaproteobacteria* | | | | | | | | Uncultured bacterium | | |  | | 2 | JF737919 | 99 |
|  | *Gammaproteobacteria* | | | | | | | | | |  | | | | 4(10) |  |  |
|  | | *Legionellaceae* | | | | | | | | *Legionella* | | | |  | 2 | FJ230896 | 96 |
|  | | *Moraxellaceae* | | | | | | | | *Psychrobacter* | | | |  | 1 | CP000323 | 99 |
|  | | Unclass. *Gammaproteobacteria* | | | | | | | | Uncultured bacterium | | | |  | 1 | EU370315 | 99 |
|  | *Deltaproteobacteria* | | | | | | | | |  | | | |  | 1(2.5) |  |  |
|  | | *Myxococcaceae* | | | | | | | | *Anaeromyxobacter* | | | |  | 1 | EU335164 | 97 |
| *Firmicutes* | | | | | | | | | | | | | | |  |  |  |
|  | *Bacilli* | | | | | | | | | |  | | | | 3(7.5) |  |  |
|  | | *Bacillaceae* | | | | | | | | Uncultured *Bacillus* | |  | | | 3 | AJ276809 | 99 |
|  | *Clostridia* | | | | | | | | |  | |  | | | 1(2.5) |  |  |
|  | | *Clostridiaceae* | | | | | | | | Uncultured *Clostridium*. Probably IRB | | | | | 1 | JF428988 | 99 |
| *Actinobacteria* | | | | | | | | | | | | | | |  |  |  |
|  | *Actinobacteria* | | | | | | | | | | | | | | 1(2.5) |  |  |
|  | | *Acidimicrobiaceae* | | | | | | | | Uncultured bacterium | | | | | 1 | FJ625358 | 97 |
| Unclassified *Bacteria* | | | | | | | | | | | | | | | 2(5) |  |  |
|  | Candidate division TM6 | | | | | | | | | Uncultured bacterium | | | | | 2 | JF428914 | 96 |
| *Chlorophyta* | | | | | | | | | | | | | | |  |  |  |
|  | *Chlorophyceae* | | | | | | | | |  | | | | |  |  |  |
|  | | *Chlamydomonadaceae* | | | | | | | | *Chlamydomonas* | | | | | 4 | AY082979 | 99 |
| *Stramenopiles* | | | | | | | | | | | | | | |  |  |  |
|  | *Chrysophyceae* | | | | | | | | |  | | | | |  |  |  |
|  | | *Chromulinaceae* | | | | | | | | *Ochromonas* | | | | | 2 | AY082982 | 99 |
| Concepción 4 m | | | | | | | | | | | | | | | | | |
| *Proteobacteria* | | | | | | | | | | | | | | |  |  |  |
|  | *Alphaproteobacteria* | | | | | | | | | | | | | | 53(74) |  |  |
|  | | *Acetobacteraceae* | | | | | | | | *Acidiphilium* IRB | | | | | 35 | JF737921 | 99 |
|  | |  | | | | | | | | *Acidisphaera* IRB | | | | | 18 | HM745451 | 99 |
|  | *Gammaproteobacteria* | | | | | | | | | | | | | | 18(25) |  |  |
|  | | *Enterobacteriaceae* | | | | | | | | *Erwinia* | | | | | 18 | JN175337 | 99 |
| Chloroplast | | | | | | | | | | | | | | | 1(1) |  |  |
|  | Unclassified *Eukaryota* | | | | | | | | | Uncultured eukaryote | | | | | 1 | HQ420121 | 99 |
| *Stramenopiles* | | | | | | | | | | | | | | |  |  |  |
|  | *Chrysophyceae* | | | | | | | | |  | | | | |  |  |  |
|  | | *Chromulinaceae* | | | | | | | | *Ochromonas* | | | | | 2 | AY082982 | 99 |
| Concepción 7 m | | | | | | | | | | | | | | | | | |
| *Proteobacteria* | | | | | | | | | | | | | | |  |  |  |
|  | *Alphaproteobacteria* | | | | | | | | |  | | | | | 10(18) |  |  |
|  | | *Acetobacteraceae* | | | | | | | | *Acidisphaera* IRB | | | | | 1 | HQ674808 | 98 |
|  |  | | | | | | | | | *Acidocella* | | | | | 3 | JF737873 | 99 |
|  | | Unclass. *Alphaproteobacteria* | | | | | | | | Uncultured bacterium | | | | | 6 | GQ500786 | 92 |
|  | *Betaproteobacteria* | | | | | | | | |  | | | | | 18(32) |  |  |
|  | | Unclass. *Betaproteobacteria* | | | | | | | | “*Ferrovum*” | | | | | 18 | DQ480476 | 99 |
|  | *Gammaproteobacteria* | | | | | | | | |  | | | | | 4(7) |  |  |
|  | | *Legionellaceae* | | | | | | | | *Legionella* | | | | | 3 | JF779686 | 95 |
|  | | *Moraxellaceae* | | | | | | | | *Psychrobacter* | | | | | 1 | AB622688 | 99 |
| *Acidobacteria* | | | |  | | | | | |  | | | | |  |  |  |
|  | *Acidobacteria* | | | | | | | | |  | | | | | 5(9) |  |  |
|  | | *Acidobacteriaceae* | | | | | | | | *Acidobacterium* IRB | | | | | 5 | FR667830 | 99 |
| *Actinobacteria* | | | | | |  | | | |  | | | | |  |  |  |
|  | *Actinobacteria* | | | | | | | | |  | | | | | 1(2) |  |  |
|  | | *Acidimicrobiaceae* | | | | | | | | Uncult. bacterium Probably IRB, IOB^4^ | | | | | 1 | FR667769 | 99 |
| Unclassified *Bacteria* | | | | | | |  | | |  | | | | | 3(5) |  |  |
|  | Candidate division TM6 | | | | | | | | | Uncultured bacterium | | | | | 3 | EU037998 | 96 |
| Chloroplast | | | | |  | | | | |  | | | | | 15(27) |  |  |
|  | Unclassified *Eukaryota* | | | | | | | | | Uncultured eukaryote | | | | | 4 | HQ420121 | 99 |
|  | | | | |  | | | | | Uncultured eukaryote | | | | | 11 | AF289158 | 91 |
| *Stramenopiles* | | | | |  | | | | |  | | | | |  |  |  |
|  | *Chrysophyceae* | | | | | | | | |  | | | | |  |  |  |
|  | | | *Chromulinaceae* | | | | | | | *Ochromonas* | | | | | 5 | AY082982 | 99 |
| Concepción 10 m | | | | | | | | | | | | | | | | | |
| *Proteobacteria* | | | | | | | | | | | | | | |  |  |  |
|  | *Alphaproteobacteria* | | | | | | | | |  | | | | | 48(28) |  |  |
|  | | | *Acetobacteraceae* | | | | | | | *Acidiphilium* IRB^3^ | | | | | 8 | JF737921 | 99 |
|  | | |  | | | | | | | *Acidiphilium* IRB | | | | | 4 | HM745451 | 99 |
|  | | |  | | | | | | | *Acidisphaera* IRB | | | | | 2 | AF376024 | 99 |
|  | | |  | | | | | | | *Acidisphaera* IRB | | | | | 8 | EF446227 | 96 |
|  | | |  | | | | | | | *Acidisphaera* IRB | | | | | 3 | AB669479 | 99 |
|  | | |  | | | | | | | *Acidocella* | | | | | 15 | JF737873 | 99 |
|  | | | Unclass. *Alphaproteobacteria* | | | | | | | Uncultured bacterium | | | | | 3 | JF737919 | 99 |
|  | | |  | | | | | | | Uncultured bacterium | | | | | 5 | GQ500786 | 92 |
|  | *Betaproteobacteria* | | | | | | | | |  | | | | | 36(21) |  |  |
|  | | | Unclass. *Betaproteobacteria* | | | | | | | “*Ferrovum*” | | | | | 36 | DQ480476 | 99 |
|  | *Gammaproteobacteria* | | | | | | | | |  | | | | | 23(13) |  |  |
|  | | | *Legionellaceae* | | | | | | | *Legionella* | | | | | 8 | FJ230896 | 96 |
|  | | | *Enterobacteriaceae* | | | | | | | *Erwinia* | | | | | 7 | JN175337 | 99 |
|  | | | *Halomonadaceae* | | | | | | | *Halomonas* | | | | | 4 | AJ551136 | 99 |
|  | | | Unclass. *Gammaproteobacteria* | | | | | | | Uncultured bacterium | | | | | 2 | EU370315 | 99 |
|  | | |  | | | | | | | Uncultured bacterium | | | | | 2 | FN870201 | 99 |
| *Acidobacteria* | | | | | | | | | | | | | | |  |  |  |
|  | *Acidobacteria* | | | | | | | | | | | | | | 15(9) |  |  |
|  | | | *Acidobacteriaceae* | | | | | | | *Acidobacterium* IRB | | | | | 15 | FR667830 | 99 |
| *Actinobacteria* | | | | | | | | | | | | | | |  |  |  |
|  | *Actinobacteria* | | | | | | | | |  | | | | | 27(16) |  |  |
|  | | | *Acidimicrobiaceae* | | | | | | | Uncult. bacterium Probably IRB, IOB | | | | | 25 | FR667769 | 99 |
|  | | | *Nocardiaceae* | | | | | | | *Rhodococcus* | | | | | 2 | JN235141 | 99 |
| Unclassified *Bacteria* | | | | | | | | | | | | | | | 3(2) |  |  |
|  | Candidate division TM6 | | | | | | | | | Uncultured bacterium | | | | | 3 | DQ499213 | 95 |
| Chloroplast | | | | |  | | | | |  | | | | | 18(11) |  |  |
|  | Unclassified *Eukaryota* | | | | | | | | | Uncultured eukaryote | | | | | 4 | HQ420121 | 99 |
|  | | | | |  | | | | | Uncultured eukaryote | | | | | 14 | AF289158 | 91 |
| *Stramenopiles* | | | | | | | | | | | | | | |  |  |  |
|  | *Chrysophyceae* | | | | | | | | |  | | | | |  |  |  |
|  | | | *Chromulinaceae* | | | | | | | *Ochromonas* | | | | | 2 | AY082982 | 99 |
| Concepción 13.5 m | | | | | | | | | | | | | | | | | |
| *Proteobacteria* | | | | | | | | | | | | | | |  |  |  |
|  | *Betaproteobacteria* | | | | | | | | |  | | | | | 6(50) |  |  |
|  | | | *Betaproteobacteria* | | | | | | | *Thiobacillus* | | | | | 1 | FN391817 | 99 |
|  | | | Unclass. *Betaproteobacteria* | | | | | | | *“Ferrovum*” | | | | | 5 | HM745412 | 97 |
| *Actinobacteria* | | | | | | | | | | | | | | |  |  |  |
|  | *Actinobacteria* | | | | | | | | |  | | | | | 6(50) |  |  |
|  | | | *Acidimicrobiaceae* | | | | | | | Uncult. bacterium Probably IRB, IOB | | | | | 1 | HM745419 | 99 |
|  |  | | | | | | | | | Uncult. bacterium Probably IRB, IOB | | | | | 5 | FR667769 | 99 |

^1^ Relative abundance of clones and percentage, ^2^ Percentage of similarity, ^3^ IRB: Iron Reducing Bacteria, ^4^ IOB: Iron Oxidizing Bacteria
